# Electro-Optical Modulation in High *Q* Metasurface Enhanced with Liquid Crystal Integration

**DOI:** 10.3390/nano12183179

**Published:** 2022-09-13

**Authors:** Ruoying Kanyang, Cizhe Fang, Qiyu Yang, Yao Shao, Genquan Han, Yan Liu, Yue Hao

**Affiliations:** 1 Emerging Device and Chip Laboratory, Hangzhou Institute of Technology, Xidian University, Hangzhou 311200, China; 2 Wide Bandgap Semiconductor Technology Disciplines State Key Laboratory, School of Microelectronics, Xidian University, Xi’an 710071, China; 3 Shanghai Energy Internet Research Institute of State, Grid 251 Libing Road, Pudong New Area, Shanghai 201210, China; 4 The Research Center for Intelligent Chips and Devices—Zhejiang Lab, Hangzhou 311121, China

**Keywords:** lithium niobate metasurfaces, bound states in the continuum, electro-optical tunning, liquid crystal

## Abstract

Electro-optical tuning metasurfaces are particularly attractive since they open up routes for dynamic reconfiguration. The electro-optic (EO) modulation strength essentially depends on the sensitivity to the EO-induced refractive index changes. In this paper, lithium niobate (LiNbO_3_) metasurfaces integrated with liquid crystals (LCs) are theoretically investigated. Cylinder arrays are proposed to support quasi-bound states in the continuum (quasi-BICs). The quasi-BIC resonances can significantly enhance the lifetime of photons and the local field, contributing to the EO-refractive index changes. By integrating metasurfaces with LCs, the combined influence of the LC reorientation and the Pockels electro-optic effect of LiNbO_3_ is leveraged to tune the transmitted wavelength and phase spectrum around the quasi-BIC wavelength, resulting in an outstanding tuning sensitivity up to Δ*λ*/Δ*V* ≈ 0.6 nm/V and relieving the need of high voltage. Furthermore, the proposed structure can alleviate the negative influence of sidewall tilt on device performance. The results presented in this work can foster wide application and prospects for the implementation of tunable displays, light detection and ranging (LiDAR), and spatial light modulators (SLMs).

## 1. Introduction

Metasurfaces have emerged as two-dimensional (2D) arrays formed by artificial subwavelength scatters [1]. The properties of the electromagnetic wave, including the amplitude, phase, and polarization, can be controlled by adjusting the size, shape, and arrangement of the scatters [2]. Thereby, the geometric patterns of metasurfaces are precisely designed to realize a wide range of applications, from beam shifters [3] or lensing [4,5], to holographic images [6] or bio-imaging, to name a few. However, a current bottleneck in the application is that their functionalities have been set in stone after the architecture and material selection. Reconfigurable metasurfaces, which could dynamically tune the optical wavefront, are crucial components in several key optical technologies, such as SLMs [7], LiDAR [8], and general light−matter interactions [9]. An immense effort has been implemented into various modulation techniques, including optical pumping [10], thermal heating [11], mechanical [12], and electrical tuning [13]. Among all these tuning mechanisms, electrical tuning is a feasible approach to achieving light−matter interaction with continuous tunability and low power consumption, which enables the metasurfaces to integrate with other optoelectronic devices [14].

To date, reconfigurable metasurfaces with EO effects have been studied using epsilon-near-zero materials [15,16], tuned carrier concentration [17], liquid crystals [18], and MEMS [19], and kilohertz-range speed modulation has been realized [20]. Lithium niobate (LiNbO_3_), with its remarkable EO properties, enables optical modulators to achieve modulation speeds of hundreds of MHz [21]. The crystal structure of LiNbO_3_ facilitates an outstanding Pockels electro-optic effect, and the refractive index can be changed by electrical voltages on the femtosecond timescale [22]. Nonetheless, conventional metasurfaces can only support relatively lower-quality factors (*Q* factors) [23] restricting their development in EO modulation. Due to the low tuning sensitivity, a high voltage is required to attain desired resonance shift for LiNbO_3_ metasurfaces (i.e., up to ±150 V). Much effort has been put into improving the EO effect. Bound states in the continuum (BICs), which can ideally confine an optical mode to a structure with no leakage, have been proposed [24]. The ultrahigh *Q* factors of the BICs lead to a significant increase in the lifetime of photons and field confinement within the mode, which could enhance the tunability of the metasurfaces [25]. On the other hand, liquid crystals, known for their unique light-modulating property, possess high sensitivity to external stimuli, such as temperature [20], machinic stretch [26], and voltage [27]. Owing to their outstanding birefringence [28], LCs are applied successively to SLMs, liquid-crystal displays, and optical switches [29]. Recently, the possibility of combining LiNbO_3_ and LCs has been demonstrated [30]. However, to the best of our knowledge, the combined influence of the LiNbO_3_ EO effect and LC reorientation has not been reported yet.

Here we numerically demonstrate a reconfigurable LiNbO_3_ metasurface integrated with LCs for dynamic electric modulation. The theoretical analysis is carried out by the finite-element method (FEM). The results show that the array of cylinders can support the quasi-BIC, which offers unique opportunities to enhance the modulation strength. Integrated with LCs, LiNbO_3_ metasurfaces achieve the purpose of dynamic modulation by applying bias voltages. Owing to the leverage of BIC resonances and the peculiarities of those two materials, the impressive modulation strength is successfully realized, which is more efficient than the unintegrated LiNbO_3_ metasurface. Furthermore, the potential problem in experiment implementation is also discussed. The proposed structure would act as a novel dynamic EO platform for holographic displays [6] and optical communications [31].

## 2. Design and Discussion

**Figure 1:**
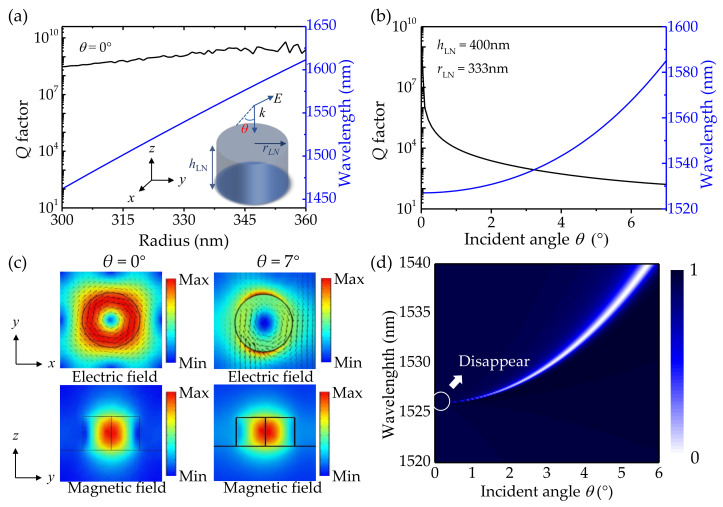
(**a**) The dependence of the *Q* factors and resonant wavelengths on the radii at *θ* = 0°. Inset: the schematic diagram of a single LiNbO_3_ cylinder. (**b**) The dependence of the *Q* factors and resonant wavelengths on the incident angle. (**c**) Electric and magnetic field distributions in the LiNbO_3_ cylinder at *θ* = 0° and 7°, respectively. (**d**) Angular-resolved transmission spectrum calculated for the periodic array with LiNbO_3_ cylinders.

In this section, a periodic structure with a cylinder array is employed to achieve BICs. The mode properties are studied by utilizing the FEM. For the sake of simplicity, a single unit is simulated in the air, as shown in the inset of Figure 1a. The radius and height of the LiNbO_3_ cylinder are denoted by *r*_LN_ and *h*_LN_, respectively. Periodic boundary conditions are used along the *x-y* directions, and perfectly matched layers are added along the *z* direction. The incident plane wave is illuminated along the *z*-axis. The ordinary refractive index of the LiNbO_3_ is set as *n*_0_ = 2.21 along the *x-y* directions, and the extraordinary refractive index is set as *n*_e_ = 2.14 along the *z*-axis. Since the periodic boundary conditions are set in the *x-y* directions, the eigenstate of the system can be given by a complex frequency, *f*. The real and imaginary parts of the complex eigenfrequency *f* represent the resonance frequency and radiation loss, respectively. The *Q* factor is calculated as follows [32]:

(1) $$Q=\frac{Re\left(f\right)}{2lm\left(f\right)}$$

In all simulations, the *h*_LN_ and the lattice constant *p* are determined to be 400 nm and 1200 nm, respectively. The dependence of the *Q* factors and resonant wavelengths of the BIC mode on different radii at normal incidence is demonstrated in Figure 1a. As the radius increases, the *Q* factor of the mode remains high (more than 10^8^). Theoretically, the *Q* factor of BICs should be infinite at normal incidence. However, due to the limitation of the simulation settings (i.e., mesh size), the *Q* factor becomes finite. Here, the ultrahigh *Q* factor is regarded as infinite [24,33,34]. With *r*_LN_ = 333 nm, the *Q* factors and resonant wavelengths of the mode for different incident angles *θ* are shown in Figure 1b. The infinite *Q* factor is obtained at the normal incidence *θ* = 0°. Then, the *Q* factor becomes finite and declines rapidly with the increase in *θ*, e.g., the *Q* factor drops to 10^2^ when *θ* increases to 7°. By contrast, this mode is sensitive to the change of the incident angle but quite insensitive to geometric change, indicating that it is a symmetry-protected BIC mode [33]. This kind of BIC is commonly realized in the symmetric structure. To gain a deeper insight into the physical mechanism of the BIC mode, the electric field and magnetic field profiles at *θ* = 0° and 7° are plotted in Figure 1c. When *θ* = 0°, the electric field and magnetic field of the symmetry-protected BIC mode are strongly confined within the cylinder. The in-plane circular current behavior of the electric field profile in one unit cell (see the top inset of Figure 1c) implies that the mode is a *z*-directed high-*Q* magnetic dipole (MD) resonance [35]. The maximum electric field transfers to the outside of the device manifesting a trend of leakage after the *θ* increases to 7° as seen in the right inset of Figure 1c. Symmetry-protected BIC mode does not radiate due to the vanishing of the coupling constants with all radiating waves. Once the symmetry is distorted, the coupling constants emerge and the ideal BIC transforms into the leaky mode with sharp resonance. The radiation powers of the MD moments are the ruling factors for these resonances [33], as can be seen from the bottom inset of Figure 1c, the distinct MD can be observed in the cylinders. To further clarify the behavior of the resonant mode, the transmission spectrum as a function of incident angle is shown in Figure 1d. The resonance narrows and fades away as the *θ* decreases to 0°, and the resonant wavelength blueshifts at the same time. When *θ* = 0°, the coupling constants with radiating waves vanish due to the exhibition of spatial symmetry, and symmetry-protected BIC is realized. The ideal BIC is a dark mode with an infinite *Q* factor and cannot be observed in the transmission spectrum. When the *θ* increases from 0° to 7°, the BIC is transformed into quasi-BIC with a decrease in the *Q* factor corresponding to the growth of radiation loss.

**Figure 2:**
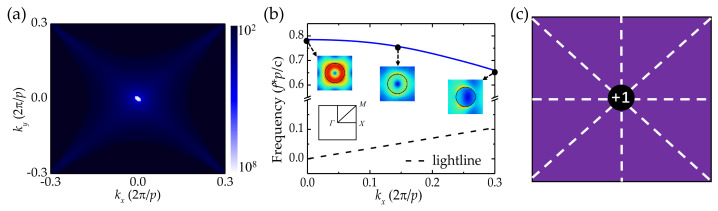
(**a**) The distribution of *Q* factor in momentum space for the LiNbO_3_ cylinder. The symmetric cylinder: *h* = 400 nm, *r* = 333 nm. (**b**) The band structure of the cylinder array. Inset: the distributions of the electric field. (**c**) The corresponding topological charge of the polarization vortices located at the *Γ* point.

To further exploit the properties of BICs, the topological configuration of a BIC is depicted in Figure 2. The *Q* factor distribution around the *Γ* point in momentum space is calculated and plotted in Figure 2a. As we can see, the BIC is generated at the *Γ* point. At this point, the light is perfectly confined in the cylinder, and the disappearance of the radiation loss results in an infinite *Q* factor. Figure 2b depicts the band structure of the periodic cylinder array. The existence of the mode above the lightline implies that the structure can support the BIC resonance at the *Γ* point of the first Brillouin zone. The robustness of the BIC is attributed to the existence of conserved and quantized topological charges, which are defined by the winding numbers of the far-field polarization vectors around the vortex centers [36]. The electric field distributions are illustrated in the inset of Figure 2b, and they become offset once *k_x_* is away from the *Γ* point. In accordance with [37], the offset directions of those field distributions along *Γ-X* can help to determine the possible topological charges at high-symmetry points. Figure 2c shows the topological nature of the symmetry-protected BIC. Because of the symmetry, the topologic charge is pinned at the center of the Brillion zone revealing one vortex with an integer topological charge of +1, corresponding to the recent observation [35]. The vortex is robust to roughness, loss, and imperfections of the structure, which is attributed to the topologic invariant in their polarization properties [36]. The topologically protected BIC could be implemented to alleviate the limitation of process tolerance.

**Figure 3:**
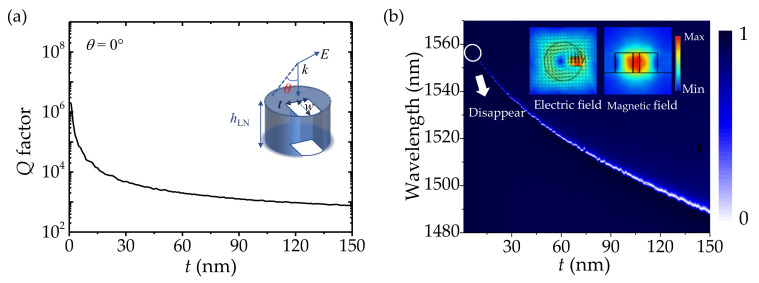
(**a**) The dependence of the *Q* factor on defect size *t* of the asymmetric LiNbO_3_ cylinder. Inset: the schematic diagram of a single asymmetric LiNbO_3_ cylinder. (**b**) Simulated transmission spectrum as a function of defect size *t*. Top inset: electric and magnetic field distributions in the asymmetric LiNbO_3_ cylinder.

A true optical BIC with an infinite *Q* factor is a mathematical abstraction, ideal lossless infinite structures or extreme values of parameters are demanded to realize it. As mentioned above, the ideal symmetry-protected BIC is sensitive to symmetric perturbation. Thus, the BIC can be transformed into the quasi-BIC mode with a high *Q* factor resonance in practice. However, the oblique incidence is unconventional for various functional metaoptics devices. Different metasurfaces composed of arrays of nanostructures with broken in-plane symmetry have emerged as a promising alternative to excite quasi-BIC resonances at normal incidence [38,39,40]. Hence, we introduce the geometric defect to the symmetric cylinder to seek the leaky mode with sharp resonance under normal incidence. The maximum width of the defect is represented by *w* and the length of the defect is represented by *t*, as shown in the inset of Figure 3a. The defect size is varied to study its influence on the *Q* factor. Since *w* is set as 200 nm, the defect size is only defined by the length *t*. The dependence of the *Q* factor on the defect length *t* is shown in Figure 3a. It could be observed that the *Q* factor is sensitive to defect size and falls quickly as *t* increases. The electric and magnetic field distributions of the asymmetric LiNbO_3_ cylinder are shown in the inset of Figure 3b. Compared to the field distributions of a symmetry-protected BIC (see the left inset of Figure 1c), the electric field extends out of the LiNbO_3_ cylinder and the intensity of the electric field located in the cylinder decreases after introducing the symmetric perturbation. The transmission spectrum as a function of defect size *t* is shown in Figure 3b. It depicts clearly that the resonance becomes broad as *t* increases and the resonant wavelength blueshifts at the same time, corresponding to the growth of radiation loss. When *t* = 0 nm, the coupling constants vanish due to symmetry, and an ideal BIC is achieved. As the defect size *t* increases from 0 to 150 nm, the ideal BIC is transformed into quasi-BIC. The relationship between the *Q* factor and defect size suggests a way to control the resonance.

**Figure 4:**
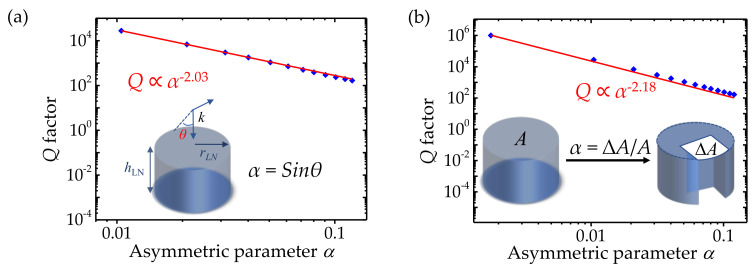
Simulated *Q* factor on the asymmetry parameter α for different design (log-log scale). Bottom Inset: the definition of the asymmetry parameter α. (**a**) Symmetric cylinder with oblique incidence. (**b**) Asymmetric cylinder with normal incidence.

To derive an analysis for the radiative *Q* factor of quasi-BIC LiNbO_3_ metasurfaces, the asymmetry parameters *α* are defined for both cases. According to [41], the relationship between *Q* and *α* of the symmetric cylinder with oblique incidence shown in Figure 4a can be expressed as *Q* ∝ *α*^−2.03^, in which *α* is defined as *α* = sin*θ*, where −2.03 is the slope of the fitting line. Owing to the different perturbations, the *α* of the asymmetric cylinder with normal incidence is defined as a function of *α* = ∆*A*/*A* (see the bottom inset in Figure 4b). *A* and ∆*A* represent the cycle area and defect area of cylinder projection in two dimensions, respectively. The behavior of the *Q* factor can be expressed as *Q* ∝ *α*^−2.18^, where −2.18 is the slope of the fitting line. It is known that the radiative losses of nearby resonances are controlled by the topological configuration of BICs. Due to the difference in the topological configurations of the two systems, the corresponding leaky modes are different, which leads to different decay rates of the *Q* factor. The robustness of the BIC is due to the existence of conserved and quantized topological charges, and those topological charges can only be generated, evoluted, and annihilated by varying structural parameters [36]. As a result, the relationship between the *Q* factor and *α* could be tuned by different systems. This mechanism provides theoretical support to manipulate the BIC and thus the behavior of the *Q* factor on *α*, which provides a platform to construct a high *Q* factor by rational design of the device structure. 

To support further applications such as SLMs and LiDAR, we apply a bias voltage to the structure for achieving EO modulation. The outstanding EO property of LiNbO_3_ makes it an ideal candidate for enhancing the tunability of optical metasurfaces. By applying an external electric field on the LiNbO_3_ layer, the refractive index of LiNbO_3_ varies due to the Pockels effect, which can be written as follows [42]:

(2) $$n=n_{0}+0.5n_{0}^{3}\gamma_{13}E$$

where *E* = *V/h*_1_ is the applied electric field. *V* and *h*_1_ represent the voltage and the thickness of the EO layer, respectively; *γ*_13_ = 10 pm/V is the EO coefficient of LiNbO_3_, and *n*_0_ = 2.21 is the ordinary refractive index of LiNbO_3_ for the zero applied electric field. The input wave is polarized along the *y* axis, thus the change of extraordinary refractive index with the applied voltage is negligible [42]. 

**Figure 5:**
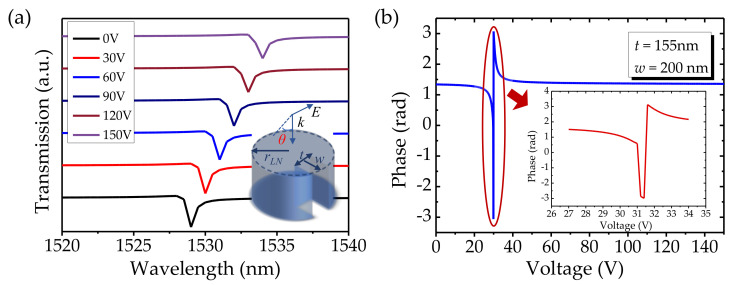
(**a**) The transmission spectra of the asymmetric cylinder for different voltages. The voltage increases from 0 to 150 V in steps of 30 V. The asymmetric cylinder: *h* = 400 nm, *r* = 333 nm, *t* = 155 nm, *w* = 200 nm, *θ* = 0°. (**b**) The transmission phase function of applied bias voltage at the operating wavelength of 1528.9 nm, *θ* = 0°.

In order to modulate the quasi-BIC resonance, we select the asymmetric LiNbO_3_ cylinder to supply voltages. Figure 5a describes the transmission property of the resonant mode for different voltages. The trough points of transmission spectra redshift with the voltages increase, and the trough points appear at the resonant wavelengths of 1529 nm, 1530 nm, 1531 nm, 1532 nm, 1533 nm, and 1534 nm at 0 V, 30 V, 60 V, 90 V, 120, and 150 V, respectively. The tuning sensitivity can be calculated as Δ*λ*/Δ*V* ≈ 0.03 nm/V. As we can see, the quasi-BIC mode manifests itself in the transmission spectrum as a pronounced Fano asymmetric line shape associated with high-*Q* factor and sharp linewidth. It is well-known that a true BIC revealing itself as a collapse of the Fano resonance results in the vanishing linewidth and the disappearance of the Fano feature in the transmission spectrum. Once the symmetry is broken, coupling between the resonant modes is governed by the perturbation, and the BIC is transformed into quasi-BIC indicating itself as a Fano line shape in the spectrum [41]. The transmission phases of the structure at the operating wavelength of 1526.9 nm are calculated and plotted as a function of applied voltage in Figure 5b. By leveraging the quasi-BIC state, a wide dynamic phase span of 2π is obtained through voltage modulation from 30 V to 33 V (see the bottom inset of Figure 5b). While the phase modulation coverage of the ITO counterpart ≈4.7 rad is obtained by voltage modulation from 0 V to 10 V, its phase modulation is enhanced by the overlap of the confined resonance and the ENZ transition of the ITO permittivity [43]. The phase modulation of the LiNbO_3_ metasurface is more substantial than that of the ITO counterpart because the quasi-BIC resonance can significantly enhance the light−matter interaction at the nanoscale, which leads to the increased lifetime of photons and strong localization of the field within the active regions of resonators. Thus, the optical path is elongated and the tunability resultant from the electro-refraction is boosted. By leveraging the quasi-BIC state, as expected, the extremely narrow spectral linewidth of the quasi-BIC with ultra-high *Q* factor can yield a substantial phase modulation in transmission. LiNbO_3_ possesses high flexibility and the amplitude and phase of its transmission can be tuned by applying different voltages while keeping the geometric parameters unchanged. This shows a way to switch channels or filter unwanted wavebands quickly and simply by changing the bias voltage [23]. This BIC-inspired metasurface can act as a suitable candidate for stronger EO modulation.

**Figure 6:**
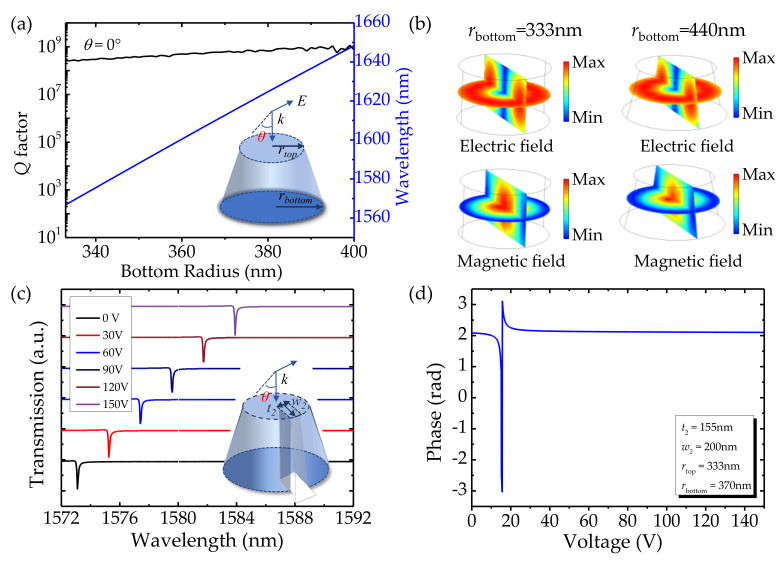
(**a**) The dependence of the *Q* factors and resonant wavelengths on the radii of the truncated cone. Inset: the schematic diagram of a single LiNbO_3_ truncated cone. (**b**) Electric and magnetic field intensity distributions at *r*_bottom_ = 333 nm (**left**) and 440 nm (**right**). (**c**) The transmission spectra of the LiNbO_3_ asymmetric truncated cone calculated for different applied bias voltages with normal incidence. The asymmetric truncated cone: *h* = 400 nm, *r*_top_ = 333 nm, *r*_bottom_ = 440 nm, *t*_2_ = 155 nm, *w*_2_ = 200 nm, *θ* = 0°. (**d**) The phase spectrum of the asymmetric truncated cone calculated for different voltages at the operating wavelength of 1574.2 nm.

Now, we discuss the potential problem in the experimental implementation of the proposed structure. It is known that LiNbO_3_ is chemically inert, so the device performance is seriously affected by the etching quality. Although tremendous efforts have been made in the field of high-quality LiNbO_3_ fabrication, such as laser ablation [44], ion beam etching [45], and focused ion beam milling (FIB) [46]. However, it is still difficult to obtain steep sidewalls in device fabrication because the reaction products always deposit on sidewalls during the etching process. Thus, the influence of non-steep sidewalls on device performance needs to be considered. To evaluate the influence of sidewall tilt on BICs, we demonstrate the evolution of the *Q* factor vs. the bottom radius of the LiNbO_3_ truncated cone in Figure 6a, where *r*_top_ and *h* are set as 333 nm and 400 nm, respectively. When the *r*_bottom_ increases from 333 nm to 540 nm, the *Q* factor retains a high value (i.e., ~10^8^). This indicates that the BIC mode is insensitive to the inclination of the sidewall. To gain a deep insight into this phenomenon, the electric and magnetic field profiles of the BIC mode with *r*_bottom_ = 333 nm and 440 nm are demonstrated in Figure 6b. The electric field and magnetic field are both strongly confined in the truncated cones. Although compared to the cylinder, the truncated cone is imperfect due to the nonideal etching, the symmetry of the structure remains. Due to topologic invariance in the polarization property, the BIC is robust to the imperfections of the structure which could alleviate the limitation of the fabrication tolerance. Thus, the truncated cone possesses similar properties to the cylinder. The influence of non-steep sidewalls on dynamic modulation is also considered. A perturbation is introduced to the truncated cone to transform the BIC to a quasi-BIC. The dimension of the perturbation is defined by the maximum width *w*_2_ and length *t*_2_, as shown in the inset of Figure 6c. The transmission spectra of the asymmetric truncated cone show the same tendency as that of the LiNbO_3_ cylinder, as shown in Figure 6c. Figure 6d shows that a wide dynamic phase span ≈ 2π of the truncated cone is also obtained through voltage modulation from 10 V to 20 V at the operating wavelength of 1574.2 nm. Such results show that the asymmetric truncated cone is as capable of optical modulation as the LiNbO_3_ cylinder. The metasurface we proposed is immune to imperfection manufacturing and possesses outstanding fabrication tolerance.

## 3. Optimization with Liquid Crystal

Although the LiNbO_3_ metasurface could be dynamically modulated, the shifts of resonances are limited due to the unsuitability of high voltage in many applications. The maximum resonance shift of quasi-BIC is around 10 nm with respect to the change of voltage. Nowadays, the integration of all-dielectric metasurfaces with nematic liquid crystals (NLCs) has been proven to be a unique strategy to obtain significant resonance tuning metasurfaces [47]. NLCs are optical uniaxial complex fluids and are known for their sensitivity to external stimuli. When a voltage is applied to the NLCs, the molecules tend to collectively reorient and align their long axes parallel to the field direction to minimize the elastic and electric energy, which causes elastic distortion of NLCs. Once the voltage is switched off, the molecules return to their original configurations because of the restoring torques provided by the elastic deformations. 

**Figure 7:**
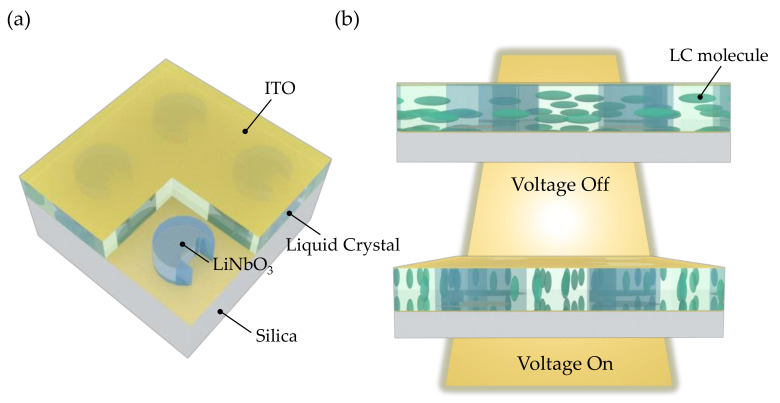
(**a**) Sketch of the LiNbO_3_ metasurface immersed in LCs. (**b**) Schematic view of the LC alignment for no applied voltage and for the case when a voltage of 70 V is applied between the two electrodes.

In order to dramatically tune the resonance positions, the LiNbO_3_ metasurface is immersed in NLCs as shown in Figure 7. (We dub this type of metasurface “integrated metasurface”). The refractive index of the silica substrate is 1.45. The indium-tin-oxide (ITO) layers are used as electrodes so that a bias voltage can be applied between the ITO electrodes. The influence of ITO layers has not been taken into account because they can be ultra-thin with a thickness of 10 nm and exhibit zero absorption at a wavelength around 1550 nm [47]. In accordance with [27], the NLCs could be represented as a homogenous medium, which possesses a large birefringence with *n*_e-LC_ = 1.7 and *n*_o-LC_ = 1.51. The *n*_e-LC_ and *n*_o-LC_ are the extraordinary and ordinary components of the NLCs refractive index, respectively. The angle between the NLC long axis and the silica plane is donated with *δ*_LC_ and can be tuned from 0° to 90° by applying the external electric field. During the angle rotation, the effective index of refraction can be written as follows [30]:

(3) $$n_{LC}=n_{o-LC}n_{e-LC}/\sqrt{n_{o-LC}^{2}\sin^{2}\left(\delta_{LC}\right)+n_{e-LC}^{2}\cos^{2}\left(\delta_{LC}\right)}$$

When there is no applied voltage, the long axes of NLCs molecules are parallel to the silica, *δ*_LC_ = 0°. By applying the external electric field, we can eventually tune the angle *δ*_LC_ to 90°, as depicted in Figure 7b. The alignment of LC molecules is preferred oriented by adding alignment layers in practical implementation. In our structure, the ITO layers can be coated with brushed layers of Nylon-6 in 2,2,2-trichlor-oethanol, which are used as the alignment layers [27]. 

**Figure 8:**
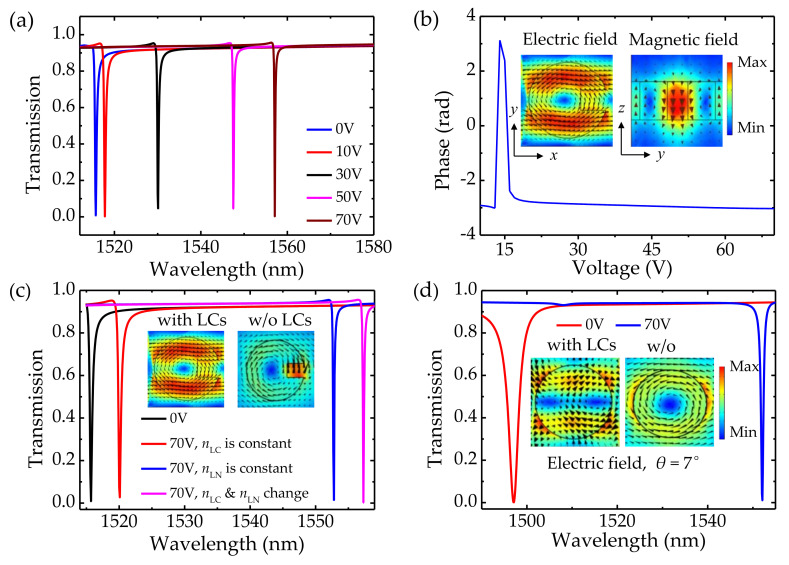
(**a**) The transmission spectra of the integrated LiNbO_3_ metasurface for different applied bias voltages. The asymmetric cylinder: *h* = 400 nm, *r* = 370 nm, *t* = 200 nm, *w* = 500 nm, *θ* = 0°. (**b**) The phase spectrum of the integrated LiNbO_3_ metasurface for different applied bias voltages at the operating wavelength of 1521 nm. The voltages increase from 0 to 70 V. Inset: Electric and magnetic field distributions of the integrated metasurface, respectively. The black arrows correspond to the electric field vectors, and the brown arrows correspond to the magnetic field vectors. (**c**) The transmission spectra of the integrated LiNbO_3_ metasurface for different kinds of refractive indices. Inset: Electric field distributions of the LiNbO_3_ cylinder integrated with and without LCs at 70 V, respectively. (**d**) The transmission spectra of the integrated metasurface with symmetric cylinders for different applied bias voltages. The symmetric cylinder: *h* = 400 nm, *r* = 370 nm, *θ* = 7°.

Figure 8a describes the transmission spectra of the integrated LiNbO_3_ metasurface for applying voltages between 0 V and 70 V. The angle *δ*_LC_ turns to 90° when the applied voltage increases to 70 V. It can be observed that the spectra redshift by 2 nm, 15 nm, 32 nm, and 42 nm when the applied voltages are 10 V, 30 V, 50 V, and 70 V, respectively. The tuning sensitivity is Δ*λ*/Δ*V* ≈ 0.6 nm/V, which is 20 times larger than that of the aforementioned unintegrated LiNbO_3_ metasurfaces. The phase spectrum of the integrated metasurface at the operating wavelength of 1521 nm is depicted in Figure 8b, in which a wide dynamic phase of 2π is attained, being the same as the unintegrated metasurfaces. Moreover, the electric and magnetic field profiles are demonstrated in Figure 8b, implying that the mode is a *z*-directed magnetic dipole resonance. To better understand the physical mechanism of the enhancement in tuning sensitivity, the electric field profiles for the integrated metasurface (left) and the unintegrated metasurface (right) at 70 V are compared, as shown in the inset Figure 8c. The maximum electric field of the unintegrated metasurface extends out of the LiNbO_3_ cylinder and concentrates in the slot port, while the maximum electric field of the integrated metasurface locates within the cylinder. The difference can be explained by the theoretical description of resonant eigenstates. According to [30], the electric field of an eigenmode can be tuned by changing of the refractive index Δ*n*, and the electric field is proportional to the overlap integral [48]:

(4) $$V_{m,\mu }=\int dVE_{m}\left(r;-k\right)\cdot \Delta n^{2}\left(r\right)E_{\mu }\left(r;k\right)$$

where, *E*_m_ and *E*_μ_ are the initial electric field and the modified eigenmodes’ electric field at frequency *ω*_m_ and *ω*_μ_, respectively, and *k* is the in-plane wave vector. When the metasurface is integrated with LCs, the change in Δ*n* is enhanced as both *n*_LiNbO3_ and *n*_LC_ are changed under applied bias. On the other hand, it is known that the light−matter interaction can be enhanced by the strong localization of the field within the resonator. In particular, the large volume of the remarkable field distributed throughout the volume of the unit cell leads to a substantial sensitivity of the spectral position to the refractive index of the resonator [30], thereby boosting the electro-refraction tunability of the integrated metasurface. In order to explore the physical origin of the resonance tuning, the contribution to resonance shift by the electric–optic effect of LiNbO_3_ and by the refractometric sensing of LCs are evaluated. The LiNbO_3_ and LCs are treated, separately, as normal media with constant refractive indices. As demonstrated in Figure 8c, the spectra redshift by 5 nm, 37 nm, and 42 nm under conditions where the *n*_LN_ changes with voltage individually, *n*_LC_ changes with voltage individually, and *n*_LN_ and *n*_LC_ change with voltage simultaneously. The tuning strength of the integrated metasurface is the sum of the LiNbO_3_ tuning and the LCs tuning. Moreover, Figure 8d describes the resonance tuning of the integrated metasurface with symmetric cylinders under oblique incidence *θ* = 7°, in which, as with the asymmetric cylinder, a wide resonance tuning is attained. The electric field profiles for the integrated metasurface (left) and the unintegrated metasurface (right) at *θ* = 7° are plotted in the inset of Figure 8d. Our simulation confirms that immersing in the LCs can help to enhance the electric field located in the cylinder. Thus, the resonance tuning of the integrated metasurface is enhanced. Many intriguing reports on EO modulation based on different metasurfaces are listed in Table 1. Due to the large birefringence in THz frequency, LC modulators have been applied in beam steering, beam shaping, and so on. However, the large pixel size may limit their ability to map sharp variations of phase. Taking the ability to reduce the pixel size into consideration, our structure intensifies the variation of the refractive index upon the electric modulation by integrating LiNbO_3_ arrays with LCs, which exhibits decent tuning sensitivity with a simpler design and thinner thickness. The results suggest that the proposed design methodology can be implemented to improve the transmission and phase modulation based on quasi-BIC in practical applications. 

## 4. Conclusions

In summary, LiNbO_3_ metasurfaces integrated with LCs are demonstrated and numerically investigated by the FEM. The simulation results indicate that the cylinder arrays support the quasi-BIC and the linear responses can be easily tailored by varying the defect size. Importantly, the introduction of LCs can strengthen the electric-optic effect. After being integrated with LCs, the impressive modulation strength has been enhanced in the near-IR wavelength range, achieving ≈ 2π phase modulation, which is more efficient compared with the ITO counterpart. Furthermore, the tuning sensitivity is up to Δ*λ*/Δ*V* ≈ 0.6 nm/V, indicating the proposed structure would be a good candidate for applications of dynamic regulation. The results provided in this work open a new avenue to achieving a novel dynamic EO platform based on LiNbO_3_.

## Figures and Tables

**Table 1:** Comparison of our results with the tuning sensitivity of different metasurfaces.

Year	Ref.	Material	Tuning Sensitivity	Thickness (w/o Substrate)
2017	[27]	Silicon/LCs	0.43 nm/V	5 μm
2018	[49]	Au/LCs	381 nm/V	5 μm
2020	[50]	BaTiO_3_	0.04 nm/V	0.2 μm
2021	[51]	JRD1:PMMA	0.031 nm/V	0.69 μm
2022	[52]	Si/LNO	0.01 nm/V	0.42 μm
2022	[42]	Al/LiNbO_3_	0.07 nm/V	0.82 μm
2022	This work	LiNbO_3_/LCs	0.6 nm/V	0.4 μm

## Data Availability

The study did not report any data.
